# Collaborative interaction points in *post*-discharge stroke care

**DOI:** 10.5334/ijic.1549

**Published:** 2014-11-06

**Authors:** Nadia Davoody, Sabine Koch, Ingvar Krakau, Maria Hägglund

**Affiliations:** Health Informatics Centre, Department of Learning, Informatics, Management and Ethics, Karolinska Institutet, Stockholm, Sweden; Health Informatics Centre, Department of Learning, Informatics, Management and Ethics. Karolinska Institutet, Stockholm, Sweden; Department of Medicine, Karolinska Institutet, Stockholm, Sweden; Health Informatics Centre, Department of Learning, Informatics, Management and Ethics. Karolinska Institutet, Stockholm, Sweden

**Keywords:** stroke, electronic tool, rehabilitation, care planning, patient participation, integrated care

## Abstract

**Introduction:**

Lack of appropriate electronic tools for supporting patient involvement and collaboration with care professionals is a problem in health care.

**Methods:**

Care and rehabilitation processes of post-discharge stroke patients were analysed using the concept of interaction points where patients, next-of-kin and care professionals interact and exchange information. Thirteen interviews with care professionals and five non-participatory observations were performed. Data were analysed using content analysis and modelling of interaction points in the patient journey.

**Results:**

Patient participation and interaction patterns vary; patients requiring home care have a passive role and next-of-kin or nurses become advocates by coordinating care on behalf of the patient, whereas patients who are able to visit primary care coordinate their own care by initiating interactions. Important categories of participation include the following: participation in care planning, in monitoring risk factors and in rehabilitation planning.

**Conclusions:**

Designing a supportive electronic tool requires understanding the interactions and patients’ activity levels at each interaction point. A tool for patients with higher activity level should support them to coordinate their own care, whereas for a less-active patient group, the tool could focus on supporting next-of-kin and care professionals in motivating, guiding and including passive patients in their care and rehabilitation processes.

## Introduction

Stroke is one of the leading causes of mortality and adult long-term disability in many countries [1], and the incidence of it has increased among adolescents and young adults in recent years [2–4]. As the number of patients suffering from multiple long-term conditions such as stroke increases significantly, the involvement of patients and their next-of-kin as well as their collaborative relationship with care professionals become a growing necessity in health care [5]. In addition, the need of improved methods for information provision to patients and their next-of-kin also increases [6–8]. Studies indicate that despite the fact that many different methods such as stroke family support services, electronic stroke education booklets and paper-based individualised information booklets for providing information to patients and their relatives have been used, the best method is still unclear [9–11]. Although increasing patient participation particularly for chronically ill patients and restructuring health care from the ‘traditional care’ to a ‘collaborative care’ wherein patients and care professionals share responsibility for problem-solving has also long been in focus [5, 12, 13], there is still limited support for a collaborative relationship between patients and care professionals. Information and communication technology has been recommended as a possible solution to this problem [14–17].

Patient participation in health care has been defined as interactions between a patient and the health care system or the health care professionals in which the patient, for example, actively provides information, asks questions and shares preferences for treatment [18]. Design of appropriate tools to support patient participation, therefore, requires an understanding of the information exchange and the interactions by investigating the engagement of patients, their next-of-kin, who can act as proxy for the patients and care professionals throughout the care processes. Previous studies, in the context of stroke, mainly focused on processes of inpatient care, remote evaluation of stroke instance and physical and cognitive rehabilitation training at home [19–23]. However, nationally in Sweden [24, 25] and internationally [26], work is underway to develop a patient-centred stroke care chain with increased focus on home rehabilitation [27]. In this study, the care and rehabilitation processes of post-discharge stroke patients have, therefore, been analysed using the concept of interaction points, i.e. where different actors (patients, next-of-kin and care professionals) in a collaborative process interact and/or exchange information. This study is part of a project aiming to design an electronic care and rehabilitation plan [28].

An electronic care and rehabilitation plan intended to be used collaboratively by stroke patients, next-of-kin and care professionals affects not only the way these actors interact with the electronic tool but also their personal relationships, work processes and environments where the tool is used. Therefore, socio-technical design [29–31] has been used as the study approach as it considers human, social and technical issues during system design. We have looked for a comprehensive picture of the way patients and next-of-kin interact with each other to understand the requirements for design of an appropriate care- and rehabilitation planning tool.

### Objective

In order to support patient participation and collaboration between post-discharge stroke patients, their next-of-kin and care professionals through information and communication technology it is crucial to explore the current care and rehabilitation processes to understand the interactions between different actors. The aims of this paper are therefore to explore current processes in post-discharge stroke care, to describe current information exchange and interaction points, and to analyse their implications for design of supportive electronic tools. Focus is hereby on involvement of patients and next-of-kin from a care professionals’ perspective.

## Theory and methods

The care and rehabilitation processes of stroke patients were explored with special focus on the interaction points between different actors in the care processes, more specifically between the patients, their next-of-kin and the care professionals. In this study, the care processes include medical treatment processes and nursing processes since they are very intertwined in post-discharge stroke care. The rehabilitation processes, however, are independent from the medical treatment and nursing processes.

### Case study setting

**Figure 1.**
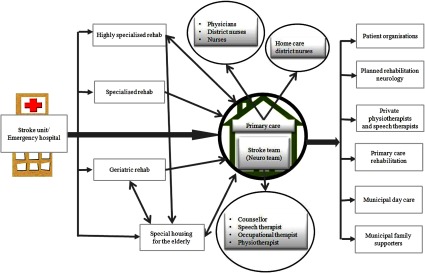
Flowchart of the stroke care chain in Stockholm County (adapted from: Lena Henricson, Karin de Haas-Ericson, and Graphics: Kjerstin Greve-Löberg, 2013-03-08).

In 2005, Stockholm County Council established a multi-professional and multi-disciplinary group of experts (including patient representatives) with the task of developing a coherent stroke care chain, particularly for long-term care. Two stroke coordinators have been responsible for identifying the continuum of stroke care, informing and creating networks between health care providers and improving routines in emergency hospitals for the transfer to the next care instance. The stroke coordinators have focused on the patient trajectory from stroke onset to subsequent primary care rehabilitation during which the patient receives care and rehabilitation at home. Figure 1 illustrates the overall stroke care chain for stroke patients in Stockholm County. The circles highlight the areas this study focuses on.

At stroke onset, the patient is sent to the emergency hospital. After acute care, depending on the patient's rehabilitation needs, the severity of stroke and the patient's physical and cognitive disabilities, the patient either receives rehabilitation in hospital and in other rehabilitation centres or at home through primary care and private physiotherapists. The primary care rehabilitation is mainly undertaken by the neuro team and private physiotherapists [32]. The neuro team consists of a speech therapist, a counsellor, an occupational therapist and a physiotherapist who provide rehabilitation with the aim of training cognitive and physical functions and adapting the housing environment. Currently, there are 20 neuro teams that are geographically evenly distributed throughout Stockholm County. Neuro teams were previously known as stroke teams, but were renamed as the teams’ mission expanded to include other brain injuries than stroke. Both names may appear in quotes, since the change took place during this study.

When stroke patients who are in need of continued care and rehabilitation efforts are discharged from the hospital or other rehabilitation centres, they receive care from various care professionals in primary care who are employed by public or private care provider organisations. Care professionals at primary care centres provide medical and nursing care. In order to follow up the patient's medication needs, a medical care plan is created in the electronic health record system by the physician together with a district nurse at the primary care centre. The neuro team creates a rehabilitation plan, to plan and follow the patient's rehabilitation process. The rehabilitation plan's main components are identifying the problem, setting goals for the rehabilitation, determining activities and follow-up.

#### Study design

An in-depth qualitative case study [33] was used as the overall research strategy. The case study was used to investigate the care and rehabilitation processes within real-life contexts and to obtain a holistic view and a deep understanding of the collaborative relationships between care professionals, patients and their next-of-kin. As post-discharge care and rehabilitation processes of stroke patients have not been clearly defined in the previous studies, this study was mainly explorative and started with a detailed investigation of the processes through different qualitative data collection methods.

#### Data collection techniques

##### Qualitative interviews

Thirteen semi-structured interviews containing open-ended questions [34] with care professionals of one neuro team and at one primary care centre were performed to explore the care and rehabilitation processes and identify the interaction points where the patients and their next-of-kin get involved throughout the processes.

A purposive selection of care professionals at a primary care centre and a neuro team in Stockholm County was performed. An important criterion was the care professionals’ experience of stroke patients and the stroke care processes. The care professionals at the primary care centre and the neuro team in this study had experience of stroke care as they received many referrals from different hospitals and rehabilitation centres and provided care and rehabilitation to several stroke patients annually. The neuro team has approximately 80 stroke patients and the primary care centre, about 50 stroke patients annually.

Initially, an interview template with pre-defined questions was provided and verified by two physicians and a district nurse to ensure the simplicity of the questions. The consulted care professionals were others than the respondents. Changes were made based on the comments and feedback received from the consultants, and two pilot structured interviews with a physician and a district nurse at the studied primary care centre were performed. Data collected from these two interviews were discussed in the research group to ensure that answers met the aim of the study. As sufficient information about the care and rehabilitation processes and the collaborative interaction points between patients and care professionals could not be obtained, a revised template for in-depth interviews was used. Unlike the previous template, all questions were not predefined and only some initial questions about the respondents’ background and the care and rehabilitation processes were defined by the research group. When a respondent was interviewed, the template was adjusted and further questions were developed based on the interviewee's responses to the previous questions. The interview consisted mostly of probing remarks for further clarification of the care processes. This change of strategy in designing the interviews and planning the questions made it possible for the researchers to explore the care and rehabilitation processes in detail and to identify interaction points throughout the processes. In total, thirteen interviews were carried out with care professionals in primary care. The first two interviews focused on the patients’ information needs and the communication between different actors in care and rehabilitation processes of stroke patients. During 11 subsequent interviews with care professionals, the participants were asked to describe their work processes with a focus on collaboration routines and patients’ participation in the care processes. The data collection continued until saturation was reached. Each interview lasted approximately 1 hour and was held at the respondent's work place. All interviews were conducted by the primary researcher (first author of this paper) and were audio recorded. From interviews, a description of the respondents’ roles and responsibilities is gathered and presented in Table 1.

In this study, we distinguish two different groups of stroke patients: non-home care and home care stroke patients. Non-home care patients refers to the patients who are living in their homes but are able to visit the health centre and receive care there and home care patients are those who require home visits by care professionals.

##### Non-participatory observations

After doing interviews with different care professionals, five non-participatory observations [34] were carried out to capture all aspects of the studied area and to complement the interviews. Participants in the observations were care professionals, patients and their next-of-kin. Table 2 provides information about the observations.

At the first observation, the home care district nurses’ work process was observed. During the first observation which was held at the primary care centre, the home care district nurses’ work process with focus on teamwork and preparation for home visits of stroke patients was observed. The second observation focused on how the home care district nurses transfer information about the nursing efforts in the electronic health record system after the home visits. The third and fourth observations were carried out at the home of two patients with the diagnosis of stroke. The researcher accompanied the district nurses to the patients’ home to observe the patient participation in the care process and the collaborative relation between patients, next-of-kin and the home care district nurses. Each home visit lasted about 5–10 minutes and the whole observation case lasted about five hours. The fifth observation was with the neuro team at a patient's home. The focus of the observation was the patient's involvement in the establishment of a rehabilitation plan together with the neuro team. During all observations, notes were taken by the primary researcher.

### Data analysis and modelling

Analysis of data was done in two stages: qualitative content analysis of interviews and observations, and modelling of interaction points in the patient journey.

#### Content analysis

All interviews were transcribed verbatim and were analysed using content analysis [33]. The transcribed interviews and notes from observations were transferred to the Nvivo 9.0 software. Nvivo was used as a tool for organising and coding the collected data. The material was worked through and analysed using an inductive approach in which codes were created from each interview and themes were identified.

The first step of analysis was detailed coding followed by more refined coding and identification of themes and categories. The analysis process was checked by two members of the research group and themes were discussed with other members in the group. Throughout the project, the collected data were validated in collaboration with other members in the research group and also through the combination of data collection methods.

#### Modelling of interaction points

The data were also analysed through modelling of interaction points in the post-discharge care processes. Interaction points can be divided into two different types: touchpoints and intersection points. In a service design, touchpoints are described as interactions between a customer and a service provider [36]. Thus in this study, touchpoints refer to interactions between patients and different care professionals who are involved in the care and rehabilitation processes of stroke patients. Intersection points, on the other hand, are defined as interactions which occur between different care professionals involved in collaborative processes [37]. In this study, we will, therefore, use the term ‘intersection’ points for care professionals’ interactions throughout the care and rehabilitation processes of stroke patients.

**Figure 2.**
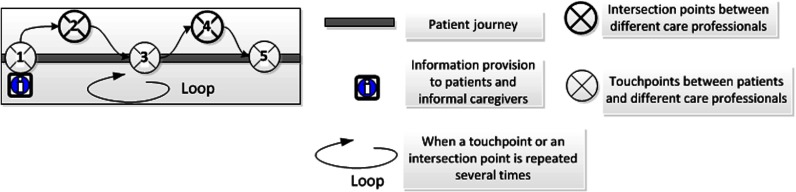
Visualisation of intersection points and touchpoints.

The care and rehabilitation processes were modelled using Microsoft Office Visio. Based on the models, the interaction points were identified throughout the processes and are presented by figures in this study. Figure 2 visualises different symbols for intersection and touchpoints.

### Ethical approval and informed consent

An ethical approval was obtained from the Regional Ethics Committee (2011/2093–31/5, 19 January 2012). A written consent was obtained (13 December 2011) from the operations manager to conduct the study at the primary care centre.

Information letters containing the purpose of the study, the procedures of the data collection and ethical considerations such as confidentiality and anonymity were provided to all participants. In addition, a short oral presentation of the study was given to the participants and an informed consent was obtained.

## Results

In the care and rehabilitation processes of stroke patients, the interaction points between patients, next-of-kin and care professionals were identified.

**Figure 3.**
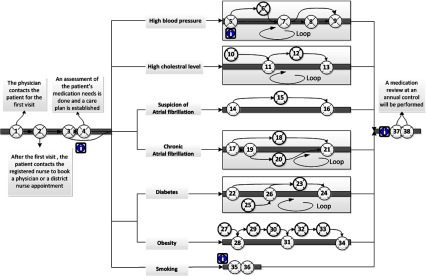
Interaction points between non-home care patients, next-of-kin and care professionals.

**Figure 4.**
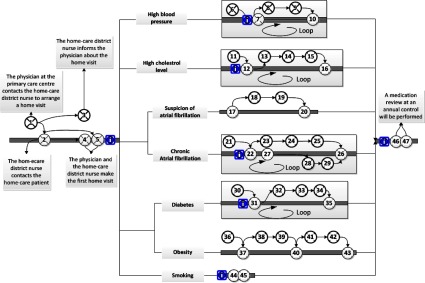
The interaction points between home care patients, next-of-kin and different care professionals.

**Figure 5.**
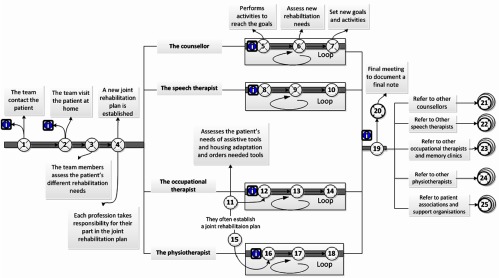
The interaction points between patients in both the patient groups, the next-of-kin and the neuro team.

Based on the patient groups, involvement of patients varies significantly. The results of this study, therefore, are presented considering the different patient groups. The results are divided into five sections: (1) interactions between care professionals at the primary care centre, non-home care patients and next-of-kin which are presented according to possible risk factors (Figure 3); (2) interactions between care professionals at the primary care centre, home care patients and next-of-kin, also presented based on risk factors (Figure 4); (3) interactions between the neuro team, the patients and the next-of-kin, which are presented, based on the rehabilitation planning process (Figure 5); (4) information provided by care professionals at different interaction points (Tables 5 and 7); and (5) different categories, based on the result of the content analysis, are presented and analysed in relation to challenges that can be addressed in the design of electronic tools. Figures 3–5 show the interaction points between different actors in the described care processes, and each numbered circle in the figure represents one interaction point.

### The interaction points between care professionals at the primary care centre and non-home care patients

Figure 3 shows the interaction points between non-home care patients, next-of-kin and different care professionals.

The care process is initiated by a referral from the hospital or the rehabilitation setting where the patient had received care for stroke and sent to the primary care centre (Figure 3). Table 3 illustrates the interaction points throughout the care processes of non-homecare patients.

#### Monitoring the risk factors

As monitoring and treatment of risk factors are essential for preventing a recurrent stroke [38], the care processes are studied considering risk factors that have been associated with increased risk of stroke incidence.

### The interaction points between the care professionals at the primary care centre and home care patients

The care process for home care patients is also initiated by a referral that is sent from the hospital or the rehabilitation setting to a primary care centre (Figure 4). Figure 4 illustrates the interaction points between home care patients, next-of-kin and different care professionals.

#### Monitoring the risk factors

The interaction points throughout the care processes for homecare patients were also studied considering risk factors that have been associated with increased risk of stroke incidence. Table 4 illustrates the interaction points throughout the care processes of home care patients.

### Information provided throughout the care processes by the care professionals at the primary care centre

Table 5 shows the information provided by care professionals at the primary care centre to patients and next-of-kin during the care processes. Non-home care patients mainly receive information about risk factors. The home care patients, however, receive information about the care professionals’ responsibilities and the care processes.

### The interaction points throughout the rehabilitation process

Figure 5 illustrates the interaction points between patients in patient groups, the next-of-kin and the neuro team.

The neuro team visits both non-home care and home care patients mainly at home. The team receives referrals either from the hospital where the patient has received care for stroke or from other rehabilitation centres. Table 6 illustrates the interaction points in rehabilitation process of patients in both groups.

### Information provision to patients and next-of-kin during the rehabilitation process

Table 7 describes the information provided by the care professionals in the neuro team to patients and next-of-kin. The main information provision includes information about the care professionals’ responsibility, support organisations in community and patient associations.

### Analysis of results and consequences for design

After identifying and describing the care processes, we continue by analysing the content of the interactions based on the interviews and the observations. Three major themes, namely, interaction patterns, patient participation and information provision and different categories are presented here. In addition, each category has been analysed in relation to challenges that can be addressed in the design and development of supportive electronic tools.

#### Interaction patterns

Patient participation and the number of touchpoints and intersection points vary throughout the care and rehabilitation processes, depending on the patients’ risk factors and their ability to manage their illness. There is a major difference between the two patient groups in the assigned responsibility for initiating interactions and between health care and patient. Patients, who suffer from several risk factors, have severe disabilities and require care at home, often have less touchpoints, and instead, there are more intersection points between different care professionals. A home care patient has a passive role and a next-of-kin and a registered nurse become advocates in the care process by actively interacting with other care professionals on behalf of the patient. For patients with fewer disabilities, who are able to visit the primary care centre, there are instead more touchpoints than intersection points. Non-home care patients coordinate their care by actively initiating the interactions with different care professionals throughout the care and rehabilitation processes. For patients, who often suffer different levels of cognitive effects, it can be quite challenging to take this coordinating and managing role themselves [39]. A supportive tool should therefore be designed considering the patient's cognitive and physical disabilities [40]. An important part for a care- and rehabilitation planning system to be used by patients would therefore be to support them in this role by, e.g. clearly presenting contact information, reminders, calendar and organisational charts.

#### Patient participation

When comparing the identified interaction points with the qualitative content analysis of the interview data, three important categories of participation throughout the care and rehabilitation process emerge: participation in care planning, participation in monitoring the risk factors and participation in rehabilitation planning.

##### Participation in care planning

Medical care planning is often a collaborative process between a physician and a nurse, as the nurse is usually the person with most direct patient contact. The medical care plan and the nursing plan are commonly created once and are continuously updated. Patient involvement in care planning is low, since care professionals consider the care plan as their working tool rather than a means for involving the patient.

The nursing plan is for the staff not for the patient. It is for the staff visiting the patient when I'm not there. The patient doesn't know for sure that I'm writing a plan, we do not tell them. But then, they are involved in e.g. dose dispensing. If we treat the wound we talk about how we do it, how this feels, etc., so they are well involved in the progress and what happens. But then when I write about it I don't think they have any idea about what I write. Some see it, because if they have wounds then the nursing plan is at the patient's home so those who come after me will always know exactly what should be done and how. (Home care district nurse 1)

The electronic care and rehabilitation plan should focus on the supporting patients and their next-of-kin where they are less involved by giving them access to an electronic care plan in which patient's medical and nursing needs are identified. In addition, the tool would increase the patients’ and their next-of-kin's participation by providing information about stroke and its consequences and risk factors. A well-informed patient is able to actively ask questions and share his/her preferences for treatment.

##### Participation in rehabilitation planning

The rehabilitation planning actively involves both the patients and their next-of-kin in discussing and identifying rehabilitation needs, goals and activities. Patients and their next-of-kin are involved in establishing and updating the rehabilitation plan.

If there is a family who wants to be involved [in the rehabilitation planning process], then they get involved in the process. (Occupational therapist)

We set goals together [we and the patient] and sometimes together with relatives also. If the patient wants the family to be involved then they will know about this [the rehabilitation plan] too, they're also taking part in this. Often there are relatives and assistants, and then we sit together but it is the patient who decides. It varies a lot how much the family are involved. (Speech Therapist)

Currently, the plan is established in the patients’ electronic health record after several visits, and a paper-based rehabilitation plan is provided to the patient at home. The paper-based rehabilitation plan is, however, sometimes lost and a copy from the neuro team is needed. To support the patients and their next-of-kin, an electronic care and rehabilitation plan should aim to give them online access to their rehabilitation plan which can be kept up-to-date and is less likely to be lost. However, being able to keep a printed version would be useful for many patients and their next-of-kin. For many patients with severe cognitive disabilities who have difficulties to manage digital interactions, an electronic plan may be less accessible than a paper-based plan. Nevertheless, studies have shown that electronic assistive tools can support the patients with disabilities in their daily life activities [41]. The physical and cognitive disabilities, however, should be considered in the design of an electronic assistive tool [39]. An electronic care and rehabilitation plan for the patients with cognitive impairments should therefore be designed to support these disabilities, e.g. by providing reminders to reduce the cognitive workload. In addition, the tool could also support the work of the next-of-kin and responsible care professionals who coordinate the care processes on behalf of the patient.

The patient has the original, I take the copy with me, but the original is not often there. Often, the patient has lost the paper then I have a copy and then I say ‘this is what we agreed on… (Speech therapist)

Depending on the patient's rehabilitation needs, the patient is entitled to receive rehabilitation from the neuro team up to one year. No planned follow-up is done after discharging from the neuro team. If a patient contacts the neuro team after discharge, the neuro team is able to provide rehabilitation for another three months, and based on the assessment of the patient rehabilitation needs, the team provides a new rehabilitation plan. Many stroke patients, however, experience a great need for continued rehabilitation/support long after they are discharged from the neuro team. A care and rehabilitation planning system should, therefore, focus on both supporting the patients and their next-of-kin in the rehabilitation process with the neuro team, as well as the period after being discharged from the neuro team by, e.g. giving access to different training and exercise programmes/videos, contact information to different care professionals and the patient's training history.

When the patient is discharged, then the process is finished, then we require a new referral, a new incidence [patient has a new stroke]. It has happened to me out of own interest that I have contacted patients to see [how the situation is]. (Speech Therapist)

##### Participation in monitoring of risk factors

Both the patient groups are involved to some extent in monitoring of risk factors. Stroke patients in both groups with different risk factors are asked to take necessary blood samples. Unlike the patients who require home care, the non-home care patients are expected to actively take necessary actions to monitor risk factors.

It becomes the patient's responsibility; we cannot continue recalling patients several times. There are patients who are happy to follow up their process themselves… There are patients, who really get involved in their treatment process and who want to know more about how to prevent a recurrent stroke, and there are other patients who do not care and it's a bit hard to force/convince them to come back to the health centre. (Physician 2)

Patient participation is about, e.g. actively interacting and taking necessary action throughout the care processes. The home care patients have a passive role in monitoring the risk factors and requiring informal caregivers and district nurses to take the responsibility in taking actions on behalf of them. The electronic care and rehabilitation plan used by this group of patients would, therefore, mainly focus on next-of-kin's and home care professionals’ needs and support them in their communication with other care professionals and in their actions for monitoring the risk factors. The electronic care and rehabilitation tool for non-home care patient would, on the other hand, aim to focus on the patients’ responsibility to coordinate their own care and take necessary actions for monitoring their risk factors by providing reminders, calendars, diary, different care professionals contact information, etc.

#### Information provision

Currently, the general information about stroke is mainly provided at the hospital where the patient has received care for stroke. Care professionals who are involved in the care and rehabilitation of post-discharge stroke patients respond to the patients’ and next-of-kin's occasional questions about stroke but do not actively provide general information about stroke and its consequences. The electronic care and rehabilitation plan, therefore, may support the involvement of the patients and their next-of-kin by focusing on provision of general information about stroke and its consequences.

The results show that the information provided by care professionals mainly includes individualised information about the risk factors and the care processes, the care professionals’ responsibilities and support organisations in community and patient associations. The information is provided in written or in oral form and depends on the patients’ risk factors, medication and rehabilitation needs.

Despite all attempts in providing information to the patients and their next-of-kin, the care professionals indicate that there is limited support for information provision to next-of-kin.

You can also improve the contact with patients and relatives. I have not had so much contact with family members but I think it is important. It [Information provision] depends on the patient's age and what resources they need. You can then provide information about what stroke is and how they [patients] can manage their illness. Families need this type of information to be able to discuss them with care professionals. (Physician 1)

“Family members may not get much support; we are there on home visits for a short while. In homecare we have no family groups; they can get a referral to a counsellor. We talk to them a lot when we're at home visits and it might count as a form of support but it is not planned.” [Homecare district nurse 1]

To support the patients and their next-of-kin in their information needs, the electronic care and rehabilitation plan would partly aim to ensure access to general information about stroke and its consequences and provide patients, next-of-kin and care professionals with possibilities to communicate through a two-way information exchange. By using such an electronic care and rehabilitation planning system the patients and their next-of-kin would have the opportunity to send their questions to the responsible care professionals and receive individualised information about, e.g. risk factors, medications and rehabilitation needs.

## Discussion

This study provides information about the involvement of the stroke patients and their next-of-kin in care processes in Stockholm and gives insight into the information provision to post-discharge stroke patients and the interaction points between different actors involved in post-discharge stroke care and rehabilitation processes.

Previous studies have focused on increasing patient participation, and a study performed in a local hospital in Sweden has showed that patients experience participation when they, e.g. receive information based on their individual needs, when they make decision based on their knowledge and needs and when they perform self-care [42]. Another study has focused on improving information provision to stroke patients and carers to facilitate the communication between them and care professionals at the acute hospital [10]. In this study, we have focused on the current care and rehabilitation processes of post-discharge stroke patients to investigate the implications that interaction between patients, next-of-kin and care professionals might have for the design of a supportive electronic care and rehabilitation plan that aims to improve patient participation and be an alternative to existing information sources.

This study was performed during the initial design phase of an appropriate care and rehabilitation planning tool using a socio-technical design approach. The collaborative interaction points from the care providers’ perspective have been studied and a comprehensive understanding of the interaction patterns between different patient groups with different care professionals has been obtained. In subsequent steps towards the design of the tool, the patients’ view on interactions with care professionals, their preferences regarding supporting tools and the technical aspects will be taken into account.

### Implications for design

In order to design such a tool that aims to improve the involvement of patients and their next-of-kin, it is of great importance to understand the interactions between patients, next-of-kin and care professionals in the care processes. To increase patients’ activity levels at each interaction point, it is important to consider how patients are involved differently depending on their capacity. The results show that the interaction patterns for home care patients consist mainly of intersection points. For non-home care patients, however, the touchpoints where the patients and care providers interact and exchange information are more frequent than intersection points. This distinction in interaction patterns is important to clarify and concretize before engaging in the design, as it will affect how the tools need to be designed to increase and support stroke patients’ participation and communication with different actors. A tool designed for non-home care patients who have fewer disabilities and higher activity levels may therefore aim to support the patients to coordinate their own care (e.g. reminders, calendar and contact information). A tool for home care patients, however, may focus on next-of-kin's and care professionals’ activities, to support them in motivating, guiding and including passive patients in their care and rehabilitation processes.

According to the interviews there is limited support for providing information to the patients and their next-of-kin and improvement is needed in information exchange between different actors involved in the care and rehabilitation of post-discharge stroke patients. We believe that an appropriate electronic care and rehabilitation planning tool could support patients, next-of-kin and care professionals’ collaborative work through a two-way health information exchange. In addition, the tool can support patient participation by giving patients access to general information about stroke and individualised information about e.g. patient's rehabilitation needs, goals and activities. As there is often a lack of resources and the time allocated for providing care for each patient is limited, it is imperative to design appropriate tools to make necessary information available for the patients and their next-of-kin. Giving patients access to necessary information through appropriate tools, will help the care professionals to mainly focus on the rehabilitation rather than allocating time for information provision. Nevertheless, there might be a problem in using the tool due to the patients’ different disabilities and the level of computer skills. As some patients may require help from the care professionals to use the tool, there is a risk that the main focus could be on handling the tool instead of providing care and rehabilitation. Therefore, finding a balance between using the tool and providing care and performing rehabilitation activities is of great importance throughout the care and rehabilitation processes. In addition, designing a tool that is as easy to use and intuitive as possible could enable the patients to use the tool independently. However, patients with severe disabilities who are not able to use electronic tools themselves can benefit from appropriate alternative solutions.

### Future work

To be able to pave the way for designing and implementing appropriate tools using information and communication technology to improve stroke care at home, there is also a need of exploring the inter-disciplinary teamwork, where the different professionals collaborate with each other to provide a good care to the patients. It is not only the collaborative relationship between the patients and the care professionals that is important, but inter-professional collaboration is also essential. Therefore, we have also studied the inter-disciplinary teamwork in home care of stroke patients and the results will be presented in a consecutive paper. To be able to design a proper supportive electronic tool, in this case the care and rehabilitation planning tool, it is also crucial to study the involvement of the patients and their next-of-kin and their collaborative relationship with the care professionals from the perspective of patients and the next-of-kin. In addition, it is imperative to study patients’ needs of supportive tools and their preferences regarding information provision throughout the post-discharge stroke care. For the detailed modelling of the care and rehabilitation processes, it is further required to verify and validate the processes, to facilitate the understanding of different steps and to identify gaps and weak links between different actors in the processes.

### Limitations

The study had to deal with a number of limitations. First, we made a conscious decision to limit the focus of the study to only a specific part of the stroke care chain and only certain actors. To gain a comprehensive overview of the current care and rehabilitation processes of post-discharge stroke patients, different care professionals (physicians, registered nurses and district nurses, home care district nurses and care professionals in a neuro team) involved in the home care processes were interviewed. The inpatient care was excluded as the earlier studies had examined the inpatient processes of stroke patients in detail [43]. In addition, we chose to not study the social care processes, but we are aware that social care often plays a major role in the care of stroke patients. Furthermore, our focus in this study has only been on the patients who were discharged from hospitals or other rehabilitation centres to their homes. Thus, patients in nursing homes or other kind of housing for elderly have not been included in the study. In addition, we did not study the interaction points from the perspective of patients and their next-of-kin. Patients’ preferences regarding information provision by care professionals was also not explored in this study. Studying these issues is of great importance for design of an electronic care and rehabilitation plan as patients and their next-of-kin might experience the interactions with the care professionals in a different way and as their desired information needs may differ from the actual information provided by the care professionals.

### Transferability

The results presented in this study are from a case study in which care processes of post-discharge stroke patients registered at a primary care centre and a neuro team was examined. The knowledge gained from this study can be transferred to other similar contexts where patients receive care and rehabilitation from care professionals in primary care. The electronic care and rehabilitation plan that will be designed based on the results from this study could also be useful for other post-discharge patients who experience similar disabilities and risk factors and are involved in the care and rehabilitation processes to some extent. However, this study cannot claim to be representative in all settings and for all patient groups as care processes and the collaborative relationship between patients, next-of-kin and care professionals might be different in other settings. Nevertheless, the results are significant for understanding the care and rehabilitation processes of stroke home care in Stockholm County, and we believe that similar interaction patterns occur in many other geographical and organisational settings too. The methods used to visualise and analyse the interactions can also be transferred to other areas where collaborative, long-term care processes are analysed.

## Conclusion

Depending on the patient's disabilities and risk factors, patient participation and the level of activity at each interaction point vary significantly. The non-home care patients need to take a great responsibility in the care process by visiting the primary care centre frequently to perform different tests, despite not having adequate tools to support them. Home care patients, however, have support from home care district nurses and often next-of-kin in communicating and coordinating care with other care providers. The differences in the activity level of patients in different groups and the variation in the interaction pattern for non-home care versus home care patients can affect the design of an electronic support system. Understanding the interaction points and the patient's activity level at each point is therefore of great importance when designing an appropriate electronic tool that supports patients, informal carers and care professionals in a collaborative process. A supportive electronic tool for home care patients can focus on supporting next-of-kin and home care professionals in their interaction with other care professionals and in involving the passive patients in their care processes, while a supporting tool used by non-home care patients mainly should focus on supporting the patients in coordinating their care.

## Figures and Tables

**Table 1.**
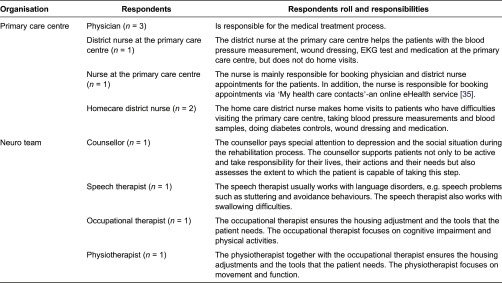
The respondents’ roles and responsibilities (based on the interviews)

**Table 2.**
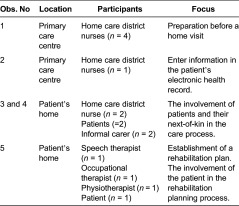
Overview of observations

**Table 3.**
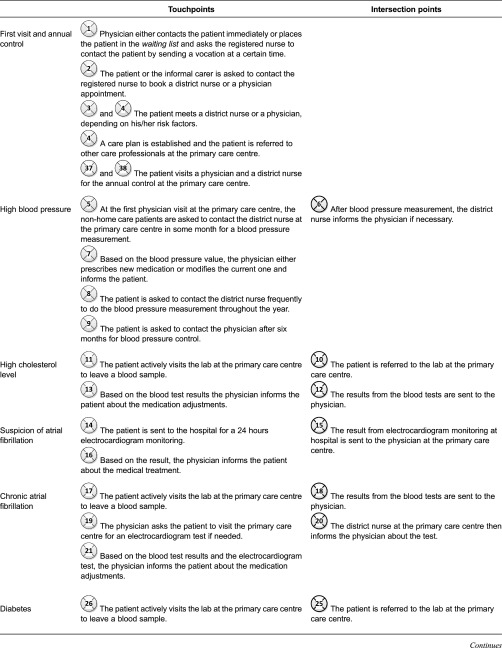

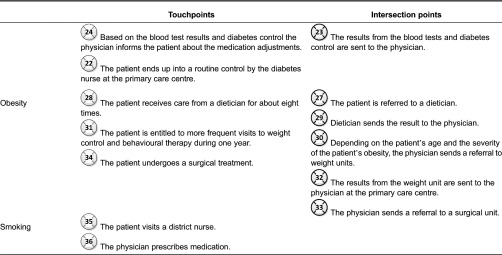
Touchpoints and intersection points in care processes of non-home care patients

**Table 4.**
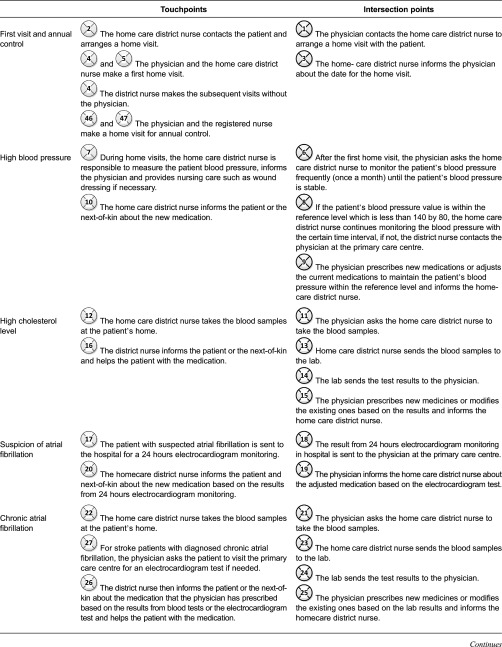

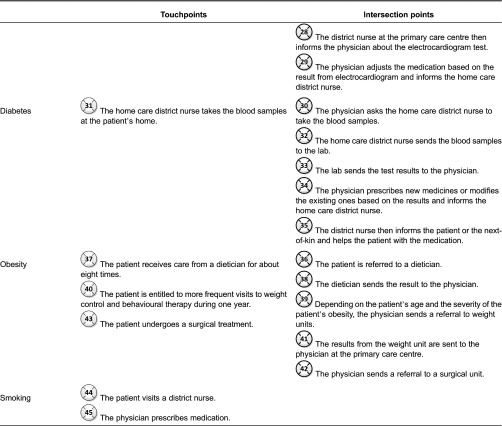
Touchpoints and intersection points in care processes of home care stroke patients

**Table 5.**
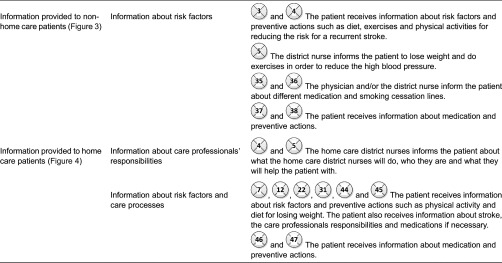
Information provided by care professionals at the primary care centre to non-home care patients and home care patients (Figures 3 and 4)

**Table 6.**
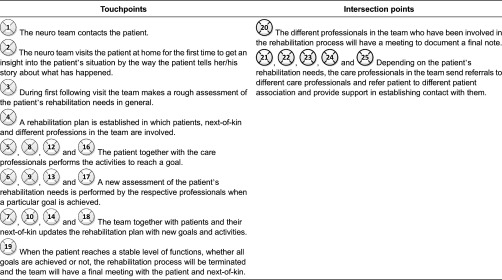
Touchpoints and intersection points in rehabilitation process

**Table 7.**
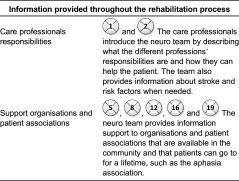
Information provision throughout the rehabilitation process
